# A retrospective study on the correction of distal arthrogryposis with a progressive extension brace

**DOI:** 10.3389/fped.2024.1385938

**Published:** 2024-04-29

**Authors:** Jiateng Zhou, Tong Zhang, Zhibo Wang, Dongdong Li, Xin Wu, Qinyuan Yu, Bin Wang

**Affiliations:** ^1^ Department of Plastic and Reconstructive Surgery, Shanghai Ninth People’s Hospital, Shanghai Jiaotong University School of Medicine, Shanghai, China; ^2^ Department of Rehabilitation Engineering, Yangzhi Rehabilitation Hospital, Tongji University, Shanghai, China

**Keywords:** distal arthrogryposis, camptodactyly, clasped thumb, brace correction, retrospective study

## Abstract

**Purpose:**

Camptodactyly, clasped thumbs, and windblown hands are distinctive features of distal arthrogryposis (DA). Current therapeutic interventions often yield suboptimal effects, predisposing patients to relapses and complications. This study explicates a corrective approach involving a progressive extension brace for the management of DA and evaluates its clinical outcomes.

**Methods:**

Between 2015 and 2023, progressive extension braces were used in 32 DA patients, with an average follow-up of 4.8 years. Patients were stratified by age into four groups: 0–1, 1–3, 3–7, and above 7 years. The correction of camptodactyly was assessed based on the total active movement (TAM) of metacarpophalangeal joints (MPJ) and proximal interphalangeal joints (PIPJ), as well as the extensor lag of PIPJ. Clasped thumb correction was evaluated by measuring the thumb-to-index finger metacarpal angle (M1M2 angle) and the degree of deviation at the first MPJ (M1P1 angle). The quality of life for the children was measured using PedsQL 4.0, while parental satisfaction was gauged using the FACE questionnaire.

**Results:**

Earlier intervention with a progressive extension brace yielded superior corrective results. Infants aged 0–1 year and toddlers aged 1–3 years achieved average TAM scores of 152° and 126° after correction; however, patients older than 3 years experienced a significant decrease in TAM with the same treatment. Infants and toddlers with DA showed improvement in the average extensor lag from 46° to 6°. The M1M2 angle increased from an average of 38° to 65°, with the M1P1 angle decreasing from an average of 43° to 5°. After the treatment, average PedsQL scores of 94.7 (parent-reported) and 89.3 (child-reported) were achieved. Among the 32 parents, 24 expressed high satisfaction, 5 expressed moderate satisfaction, and 3 expressed fair satisfaction.

**Conclusion:**

The early, progressive, and consistent use of an extension brace significantly improved joint mobility and corrected camptodactyly and clasped thumbs. It can be an effective approach to addressing hand deformities in patients with DA.

## 1 Introduction

Distal arthrogryposis (DA) was defined in 1996 as a group of heterogeneous syndromes characterized by congenital arthrogryposis affecting at least two body regions. It predominantly affects the extremities with varying degrees of involvement in proximal joints while excluding primary neurological disorders and muscular diseases that impair limb functions (1, 2). As a common subtype of arthrogryposis multiplex congenita (AMC), second only to amyoplasia, the exact incidence of DA remains unclear. A study conducted in Sweden identified approximately one-fifth of 131 AMC cases as DA (3).

The most common and functionally impactful deformities of DA involve joint contractures of the fingers, which primarily encompass camptodactyly of multiple fingers, clasped thumbs, and windblown hands (4). The reconstruction of hand function is crucial for the daily lives of individuals with distal arthrogryposis.

Camptodactyly is almost ubiquitous in all patients diagnosed with distal arthrogryposis (5). Its severity varies and can affect multiple fingers bilaterally, leading to reduced finger mobility and limited extension for grasping activities (6). Patients with ineffective conservative treatment, functional impairment, or proximal interphalangeal joint (PIPJ) flexion angles exceeding 50°–60° are generally considered for surgical intervention (7). However, due to the involvement of multiple joints in DA, current surgical methods often fail to completely correct camptodactyly and clasped thumbs, resulting in recurrence in approximately one-fourth to one-half of patients (8, 9).

While many clinicians advocate early manipulation, plaster casting, and brace interventions for correcting DA, the lack of precise studies on the timing of intervention and standardized operation procedures has resulted in unsatisfactory outcomes. This could lead to painful treatment processes, a high likelihood of relapse, and potential complications such as joint stiffness and skin breakdown.

We report a retrospective study evaluating the clinical efficacy of a progressive extension brace for correcting DA.

## 2 Patients and methods

### 2.1 Patients’ information

This retrospective study included 32 patients diagnosed with DA who underwent brace treatment between January 2015 and January 2023. Supplementary Table S1 provides demographic information about the patients. All patients exhibited flexion contractures at multiple finger joints, with amyoplasia and other primary neurological or muscular disorders ruled out through history taking and physical examination based on published classification (10–12). They subsequently underwent progressive extension brace treatment and were followed up for over 2 years. Informed consent was obtained from all parents/guardians of the patients included in this study. Ethical approval for this study was granted by the ethics committee of the Shanghai Ninth People's Hospital, affiliated to the Shanghai Jiaotong University School of Medicine.

### 2.2 Brace correction strategy

**Figure 1:**
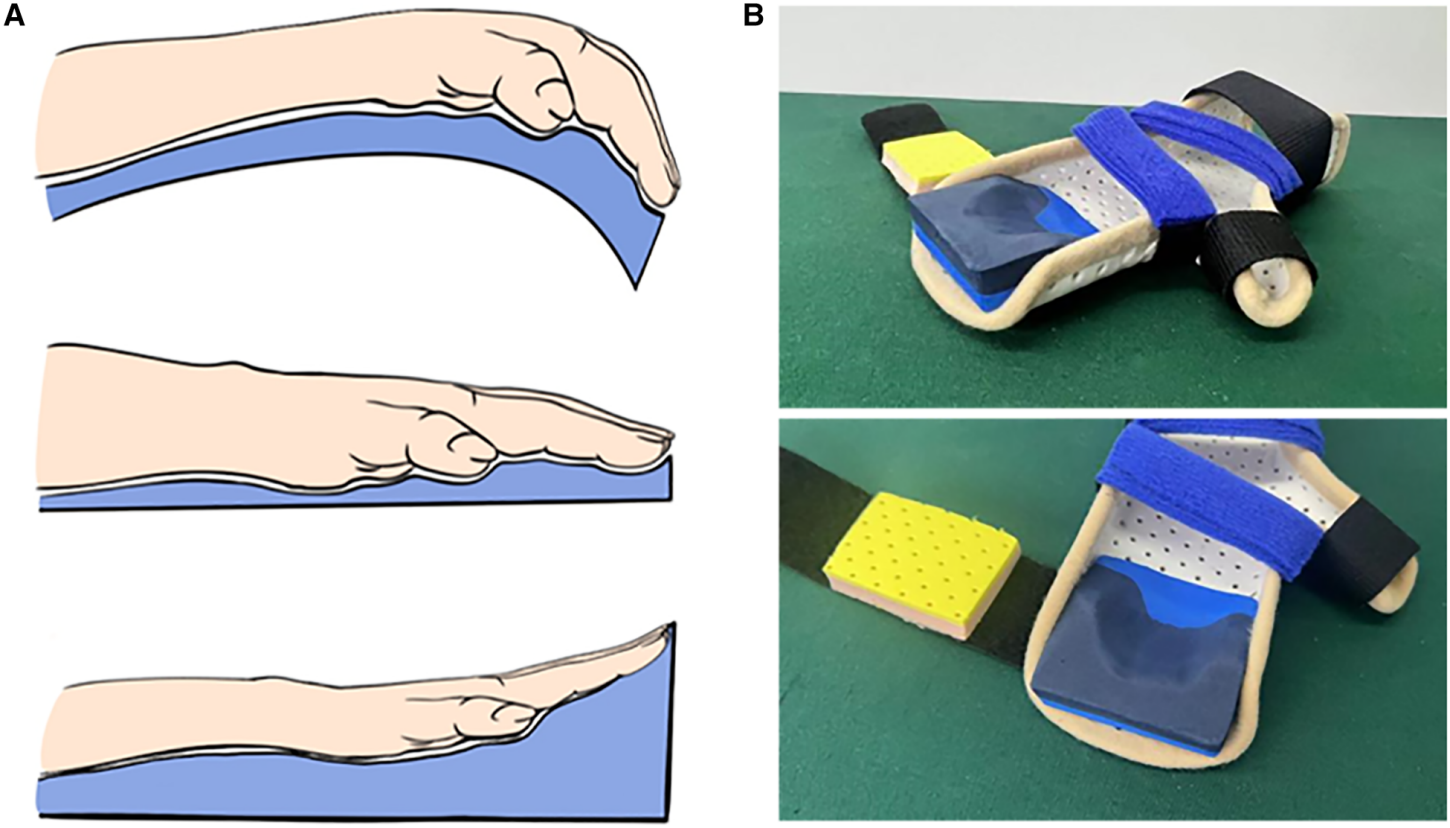
Progressive extension brace. (**A**) Schematic description of brace correction. (**B**) Physical representation of the progressive extension brace.

We propose a clinical strategy for correcting DA using a progressive extension brace. The brace employs the method of static progressive stretching with low strength over a long time, whereby soft tissues elongate with stress relaxation and creep, transitioning from elastic deformation to plastic deformation. The use of the brace follows three principles: progressive extension to rectify camptodactyly, maintaining an open web space to straighten the clasped thumb, and continuous application throughout development. Patients with DA can commence braces from infancy, starting at around 1-month-old. It is recommended that children wear the braces during both night-time and daytime sleep for 8–12 h per day, whereas they engage in joint activities and hand function training during the remaining time. Continuous wear and regular follow-up visits facilitate ongoing adjustments of the brace, which transitions from a flexed to an extended position and eventually to an overextended position (Figure 1). Each phase is maintained for 2–3 months after achieving the desired adjustment; afterward, the brace is maintained throughout the hand developmental period (up to age 12).

### 2.3 Clinical evaluation

To assess the clinical effects of early intervention on the correction of these hand deformities, we categorized the patients into four groups based on the age at treatment: 0–1 year (*n* = 11), 1–3 years (*n* = 9), 3–7 years (*n* = 7), and above 7 years (*n* = 5). The total active movement (TAM) of metacarpophalangeal joints (MPJ) and PIPJ and the extensor lag of PIPJ of the affected fingers were recorded using a goniometer to evaluate camptodactyly. The thumb-to-index finger metacarpal angle (M1M2 angle) and the degree of deviation at the first MPJ (M1P1 angle) were measured with the first web space open to assess the clasped thumb (13). The PedsQL 4.0 Generic Core Scales were used to assess the quality of life patients. Parental satisfaction with brace correction was evaluated using the FACE questionnaire (14). Results were expressed as mean ± SD. Between-group comparisons were conducted using unpaired *t*-tests, and a *p*-value < 0.05 was considered statistically significant (*p < 0.05, **p < 0.01, and ***p < 0.001). All statistical analyses were performed with GraphPad Prism 9 software.

## 3 Results

### 3.1 Clinical evaluation of progressive extension braces

**Figure 2:**
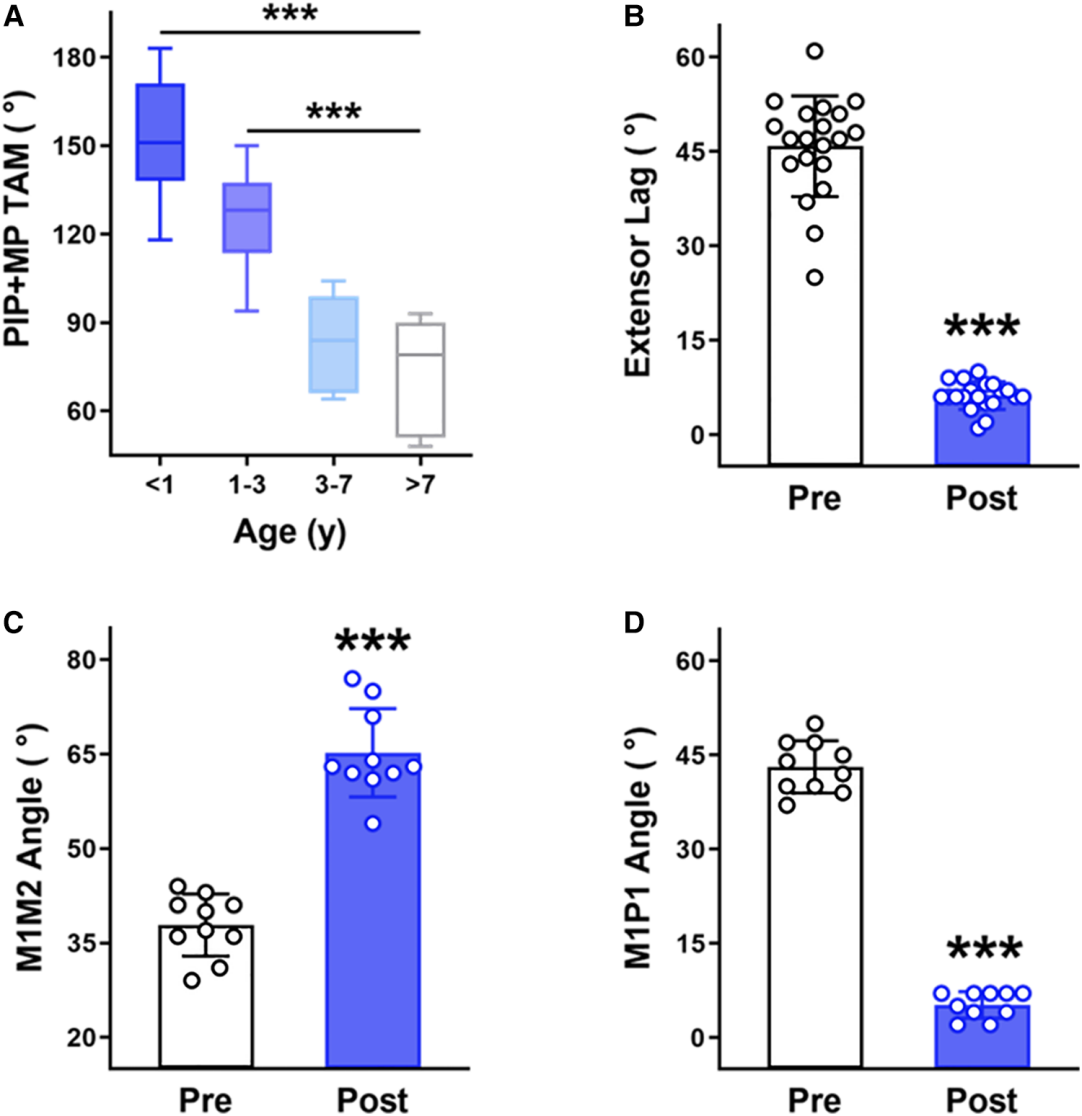
Clinical evaluation of a progressive extension brace. (**A**) The TAM of PIP and MP joints after brace correction in DA patients of different age groups. (**B**) The PIP joint extensor lag before and after brace correction in the I&T group of DA patients. (**C**) The M1M2 angle before and after brace correction in the I&T group of DA patients with clasped thumbs. (**D**) The M1P1 angle before and after brace correction in the I&T group of DA patients with clasped thumbs. ***p- < 0.001, unpaired *t*-test.

Thirty-two DA patients underwent progressive extension brace treatment with an average follow-up of 4.8 years (4.8 ± 1.2 years). Patient compliance was very good, with no occurrence of skin breakdown or related complications. We found that the most favorable outcomes in progressive extension brace correction were observed in infants (under 1 year), who achieved an average TAM of up to 152° after correction. Toddlers (1–3 years old) with DA also demonstrated significant improvement, reaching an average TAM of 126°. They are rated as excellent and good according to the Strickland criteria. Patients aged 3–7 years old and above 7 years old achieved average TAM scores of only 83° and 72°, respectively, rated as poor based on the Strickland criteria (Figure 2A, Supplementary Table S2). These findings reveal superior brace treatment outcomes in DA patients when initiated during infancy or early childhood and underscore the imperative for early intervention.

By comparing the changes in the extensor lag of infants and toddlers with DA (I&T group) before and after brace correction, we observed a decrease from an average of 46° to 6° after treatment, demonstrating effective rectification of camptodactyly (Figure 2B).

The clinical presentation of a clasped thumb primarily involves a narrowed first web space. A smaller M1M2 angle and a larger M1P1 angle correspond to a more severely clasped thumb. In the I&T group, 10 cases exhibited pronounced clasped thumbs. Brace correction significantly reduced the M1P1 angle (decreased from an average of 43° to 5°) and increased the M1M2 angle (increased from an average of 38° to 65°), demonstrating effective correction of the clasped thumb (Figures 2C,D) in the I&T group.

In addition, DA patients achieved average PedsQL scores of 94.7 (parent-reported) and 89.3 (child-reported) after brace correction. Specifically, the I&T group attained average scores of 95.7 (parent-reported) and 90.2 (child-reported) after receiving brace intervention, indicating a quality of life close to that of normal children (Supplementary Table S3). Among all the parents, 24 expressed high satisfaction with the treatment for their children, 5 reported moderate satisfaction, and 3 expressed a fair outcome (Supplementary Table S3).

### 3.2 Typical cases of progressive extension brace correction

**Figure 3:**
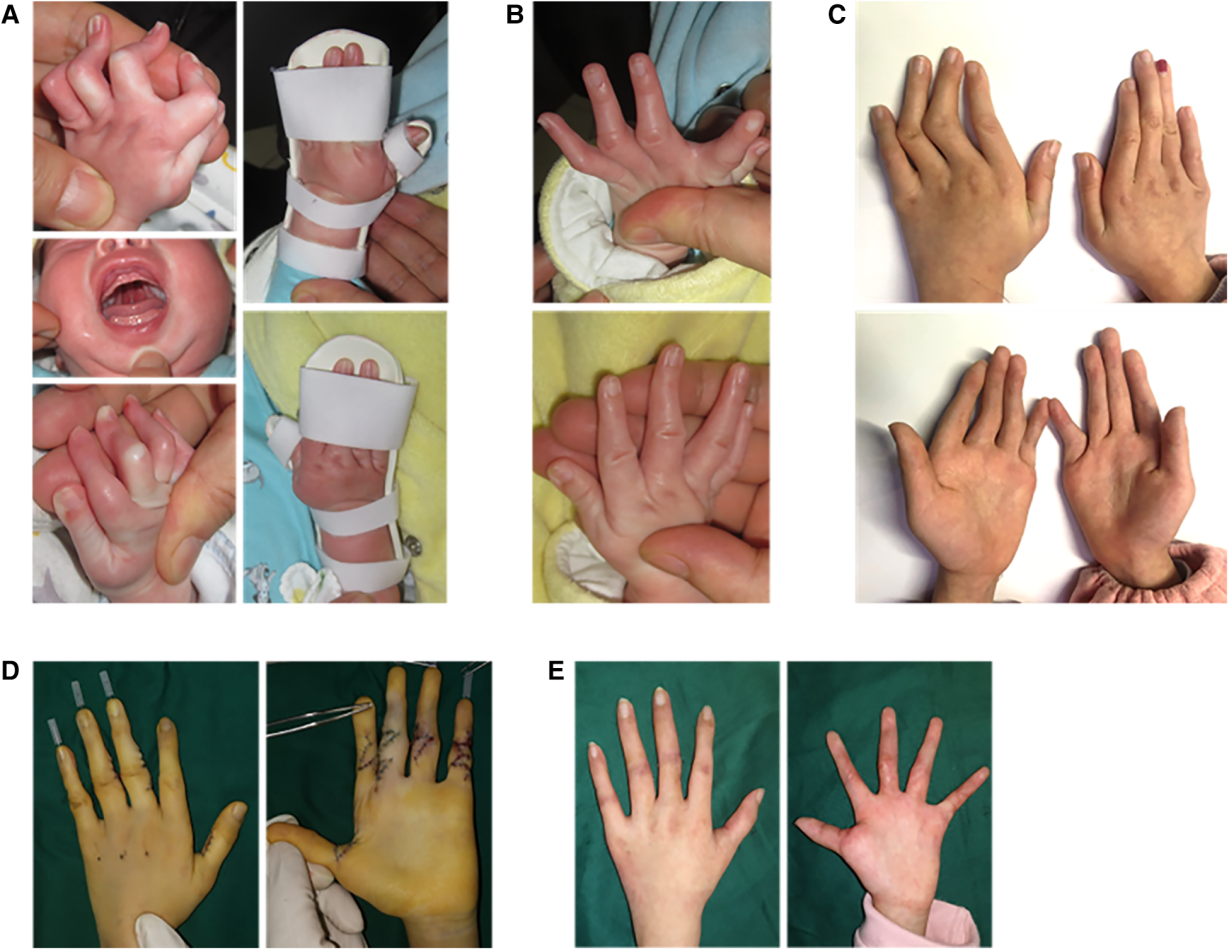
A 2-month-old female patient with distal arthrogryposis in both hands was treated with a progressive extension brace and surgery. (**A**) The patient presented severe bilateral camptodactyly along with a cleft palate and underwent progressive extension brace treatment for correcting the hand deformities. (**B**) The appearance of the hands after 1 month of brace correction. (**C**) The appearance of the hands after 5 years of brace correction. (**D**) Left-hand corrective surgery. (**E**) The appearance of the left hand 3 months after surgery.

A female DA patient, 2 months old with a cleft palate, presented a clasped thumb and severe camptodactyly with the extensor lag exceeding 90° in multiple fingers (Figure 3A). A remarkable reduction in the extensor lag was observed after just 1 month of progressive extension brace correction (Figure 3B), and the brace use was continued to prevent subsequent contracture recurrence. Partial deformities in the PIP joints appeared in the patient after 5 years of treatment due to rapid finger growth and development (Figure 3C). However, thanks to the correction of joint contractures by the brace, we only needed to perform the double-opposing Z-plasty with a Y to V advancement on the little finger and Z-plasty on other fingers to complete the surgical correction without the need for additional skin grafting on the palm (Figure 3D, Supplementary Figure S1). At the 3-month postoperative follow-up, remarkable improvement in the patient's left fingers was observed (Figure 3E).

**Figure 4:**
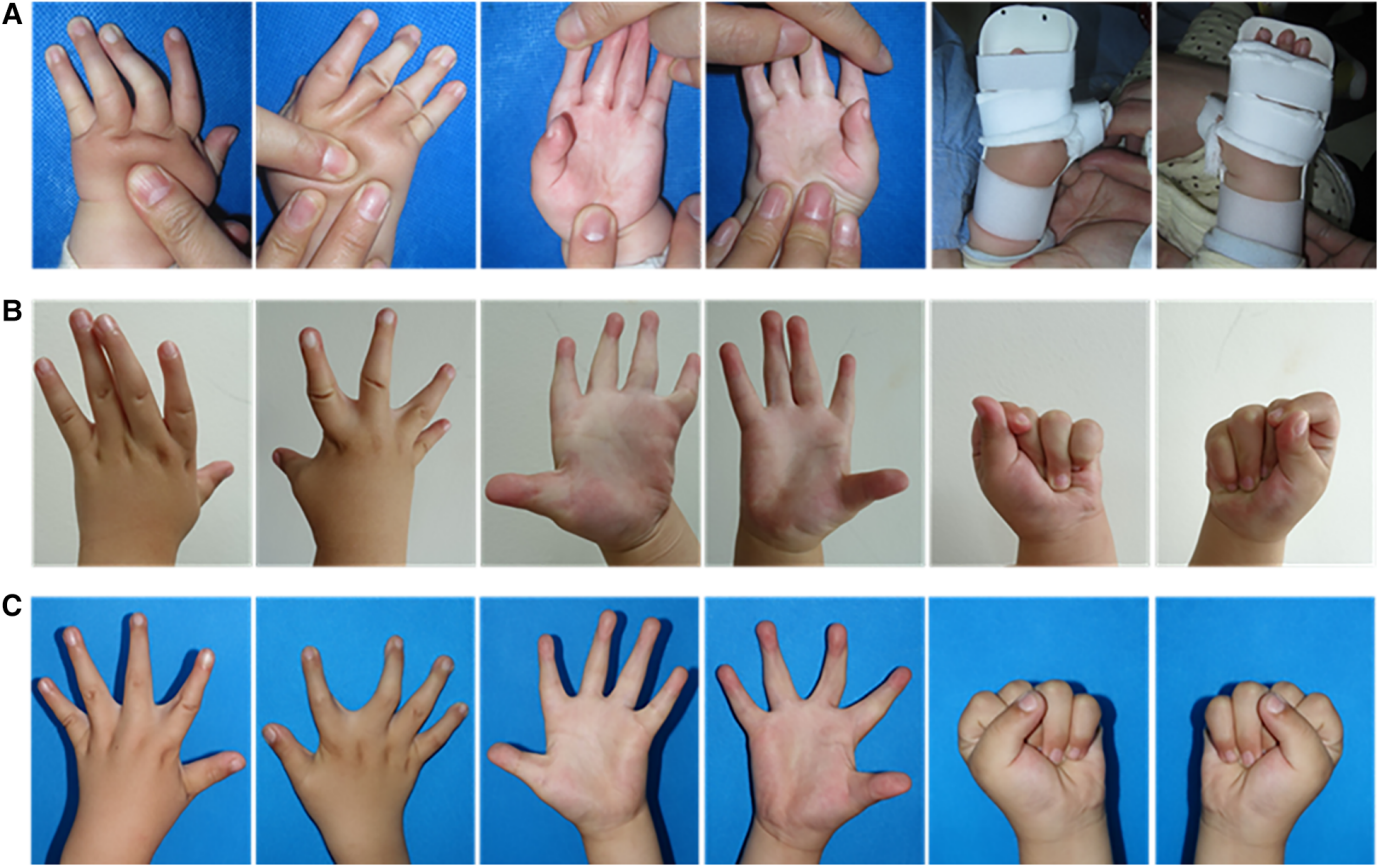
A 6-month-old male patient with distal arthrogryposis in both hands was treated with a progressive extension brace. (**A**) Correction of bilateral camptodactyly with the associated clasped thumb using a progressive extension brace. (**B**) The appearance and flexion-extension function of the hands after 10 months of brace correction. (**C**) The appearance and flexion-extension function of the hands after 30 months of brace correction.

**Figure 5:**
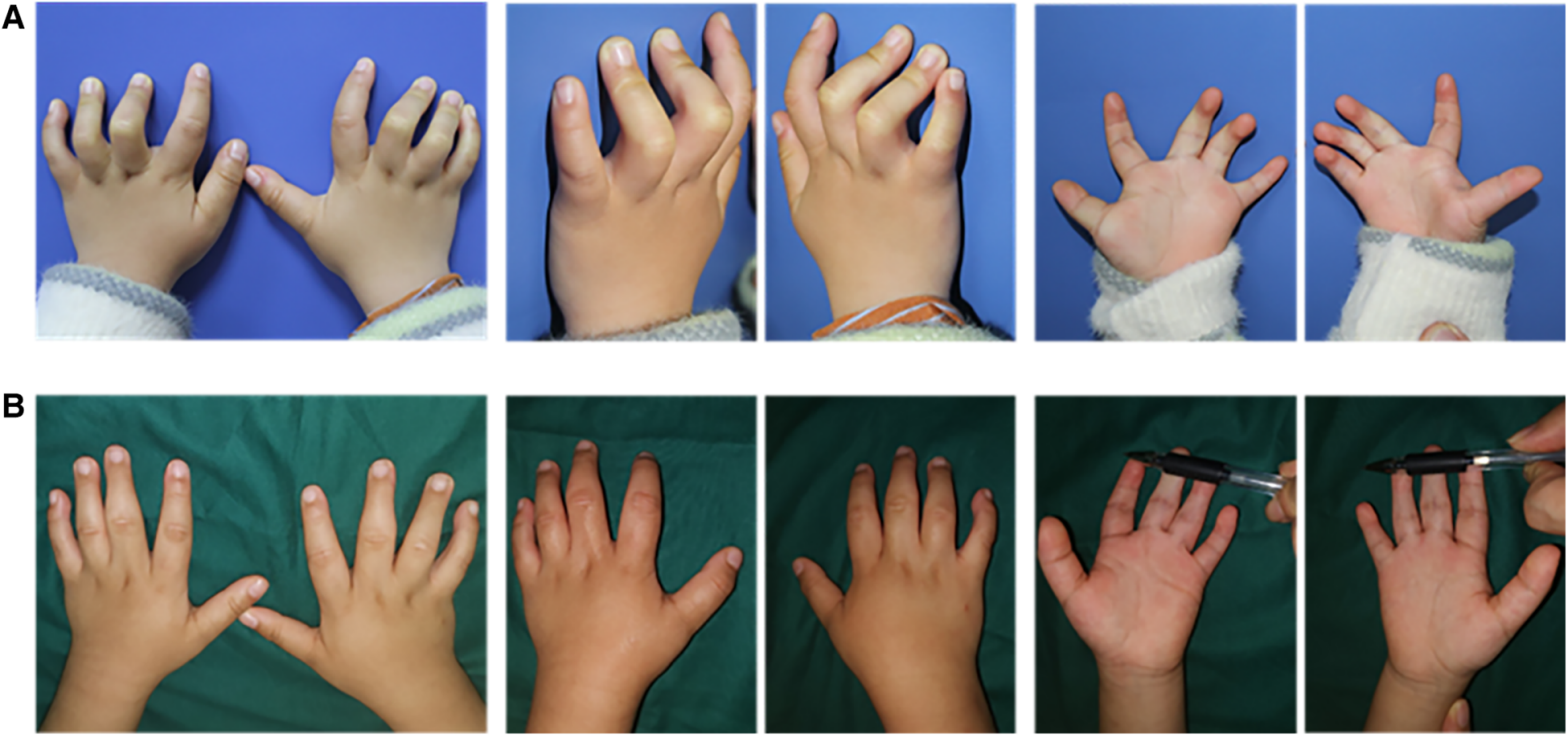
A 2-year-old male patient with distal arthrogryposis in both hands was treated with a progressive extension brace. (**A**) Bilateral camptodactyly. (**B**) The appearance of the hands after 8 months of brace correction.

In a DA patient with a clasped thumb and ulnar deviation deformities (Figure 4A), the correction of camptodactyly was noticed after 10 months of brace treatment, although some degree of thumb adduction and ulnar deviation persisted (Figure 4B). Subsequently, after 30 months of continuous brace use, significant improvements were observed in finger flexion and extension function, the first web space dimensions, and ulnar deviation. Both hands achieved nearly normal function without the need for surgical intervention (Figure 4C). Another toddler with distal arthrogryposis in both hands also exhibited favorable corrective outcomes after progressive extension brace treatment (Figure 5).

## 4 Discussion

The clinical phenotype of distal arthrogryposis patients most commonly presents camptodactyly, clasped thumbs, and windblown hands. Despite the advocacy of many clinicians for early traction, splinting, and brace correction, there is a lack of systematic studies on the timing and methods of intervention. Due to the absence of standardized treatment protocols, many patients miss the optimal intervention window, which could result in extensive contractures of the palm, tendons, and skin, and require an extensive surgical intervention at a later stage. In severe cases, heightened vascular tension is high can lead to circulatory disturbances at surgical release.

In this study, we propose a strategy utilizing a progressive extension brace to correct hand deformities in DA patients and demonstrate its consistent clinical efficacy. Camptodactyly in DA patients involves a wide range of tissues, including the flexor digitorum superficialis, lumbrical muscles, volar plate, and generic retinaculum cutis. Continuous and progressive use of the brace gradually relaxes these soft tissues. Through repeated follow-up visits, we systematically adjust the degree of brace extension and effectively rectify hand deformities while significantly enhancing patient compliance without any complications. Previous studies have discussed the effect of brace correction on camptodactyly (6); however, the impact of intervention age needs to be emphasized to ensure the best treatment outcomes. Some researchers advocate for early surgical intervention in children where tissue adhesions due to camptodactyly are not severe to prevent secondary changes and suboptimal surgical outcomes resulting from delayed treatment (15). Our study findings indicate that the earlier the intervention age for brace treatment, the better the outcomes will be. Satisfactory clinical results are often achieved in infants and toddlers (0–3 years), whereas the effectiveness of brace correction significantly decreased after the age of 3. Furthermore, we recommend continuous brace application throughout the child's hand developmental period to maintain corrective effects and reduce recurrence rates. This approach simplifies surgical treatments later and reduces the need for skin grafting for camptodactyly and clasped thumbs that cannot be completely corrected by a progressive extension brace.

A clasped thumb is characterized by thumb supination, adduction, and flexion contracture, often accompanied by a narrowed first web space and weakness of the extensor muscles. Patients with clasped thumbs can adapt to certain functional activities, but their grip function is significantly impaired due to the thumb's inability to oppose the fingers (16). We found that a progressive extension brace can widen the narrowed first web space, promote extensor tendon development, and establish a solid foundation for later corrections. For clasped thumbs that are challenging to fully rectify, we often achieved satisfactory outcomes through muscle endpoint repositioning and local skin flap transposition, which prevented the extensor indicis proprius transfers.

## 5 Conclusion

Early, progressive, and continuous use of an extension brace significantly improves joint mobility and corrects camptodactyly and clasped thumbs. It is an effective approach to rectify hand deformities in DA patients.

## Data Availability

The original contributions presented in the study are included in the article/**Supplementary Material**, further inquiries can be directed to the corresponding authors.
