# Supplementary material for: A retrospective study on the correction of distal arthrogryposis with a progressive extension brace
Source: Front Pediatr. 2024 Apr 29;12:1385938. doi: 10.3389/fped.2024.1385938 (PMC11089123; doi:10.3389/fped.2024.1385938)
Supplement: Supplementary file 1 [file Datasheet1.pdf]

Supplementary Material

Supplementary Figure 1: **The surgical flaps design of the DA patient in Figure 3**

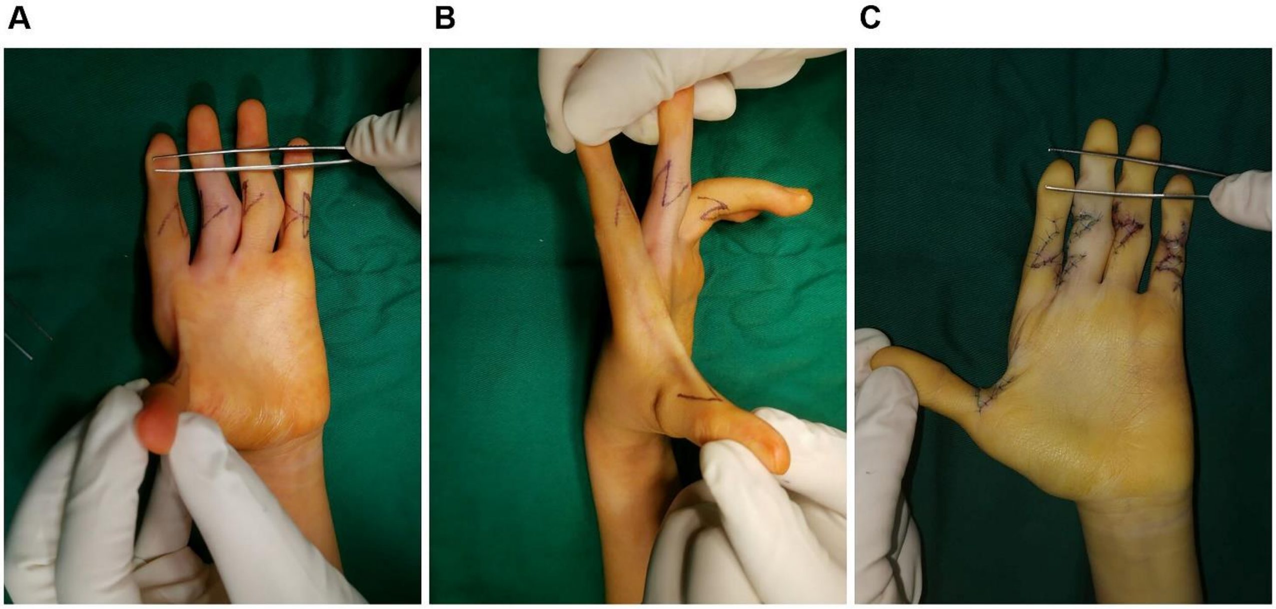

Supplementary Figure 2: The surgical flaps design of the DA patient in Figure 3. A: The planned flaps view from the palmar aspect. B: The planned flaps view from the radial aspect. C: Postoperative appearance of the palm.

Supplementary Table 1. Demographic information of DA patients

Patients information	n=32
**Gender (male/female)**	17/15
**Average age**	3.4±3.2 years (2 months - 10 years)
**Hands (left/right/both)**	7/5/20

**Follow-up time (years)**	4.8±1.2 (2 - 7)

Supplementary Table 2. Clinical evaluation of progressive extension brace

Measurement	Pre (°)	Post (°)
**Camptodactyly:**		
TAM (PIP+MP)		
< 1 year	85±14	152±21
1-3 years	72±11	126±17
3-7 years	69±9	83±16
>7 years	65±9	72±20
Extensor Lag (PIP)	46±8	6±2
**Clasped Thumb:**		
M1M2 Angle	38±5	65±7
M1P1 Angle	43±4	5±2

Supplementary Table 3. PedsQL score and parental satisfaction

Age	0-3 years	＞3 years	All
**PedsQL:**			
Parent	95.7±2.6	93.1±3.9	94.7±3.3

Child	90.2±5.1	87.8±3.7	89.3±4.7
**Satisfaction Level (1-5):**			
5	18	6	24
4	2	3	5
3		3	3
